# Supplementary material for: Electrodeposition of Ti-Doped Hierarchically Mesoporous Silica Microspheres/Tungsten Oxide Nanocrystallines Hybrid Films and Their Electrochromic Performance
Source: Nanomaterials (Basel). 2019 Dec 17;9(12):1795. doi: 10.3390/nano9121795 (PMC6955696; doi:10.3390/nano9121795)
Supplement: Supplementary file 1 [file nanomaterials-09-01795-s001.pdf]

Supplementary Materials

Electrodeposition of Ti-Doped Hierarchically Mesoporous Silica Microspheres/Tungsten Oxide Nanocrystallines Hybrid Films and Their Electrochromic Performance

Ya Song [1], Zhiyu Zhang [1], Lamei Yan [2], Ling Zhang [3], Simin Liu [3], Shaowen Xie [1,3], Lijian Xu [3,4] and Jingjing Du [1,4]*

[1] College of Packaging Materials and Engineering, Hunan University of Technology, Zhuzhou 412008, China; sy18798448247@163.com (Y.S.); zzhiyu12@126.com (Z.Z.); 13502431554@163.com (S.X.)

[2] School of Digital Media and Design, Hangzhou Dianzi University, Hangzhou 310018, China; ylm@hdu.edu.cn

[3] Hunan Key Laboratory of Biomedical Nanomaterials and Devices, College of Life Sciences and Chemistry, Hunan University of Technology, Zhuzhou 412007, China; lingzhang645@126.com (L.Z.); 18890229883@163.com (S.L.); xlj235@163.com (L.X.)

[4] National & Local Joint Engineering Research Center of Advanced Packaging Materials Developing Technology, Hunan University of Technology, Zhuzhou 412008, China

*Correspondence: 10668@hut.edu.cn; Tel.: +1-815-382-0923

Contents

Fig. S1. EDXS pattern of the THMSs.

Fig. S2. Cyclic voltammograms of WO_3, THMS/15WO_3 and HMS/15WO_3 films at a scan rate of 50 mV s^{-1}.

Fig. S3. CV curves of (a) pure WO_3, (b)THMS/5WO_3, (c)THMS/10WO_3, (d) THMS/15WO_3 and (e) THMS/20WO_3 hybrid films at different scan rates, (f) Plots of peak current density versus square root of scan rate for pure WO_3 and THMS/WO_3 films.

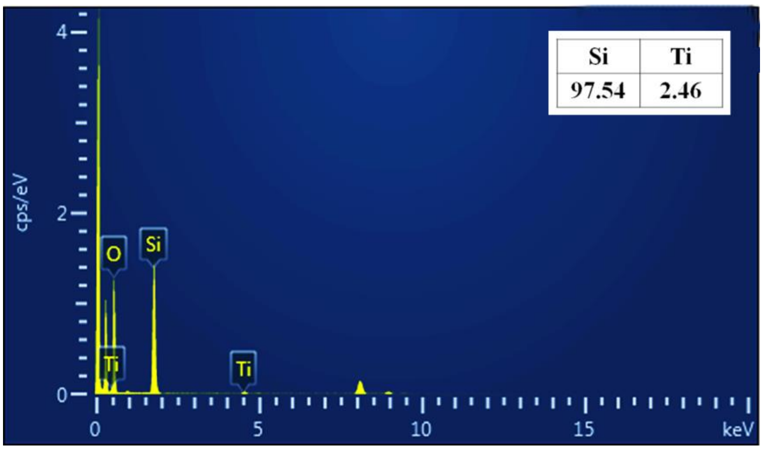

Fig. S1. EDXS pattern of the THMSs.

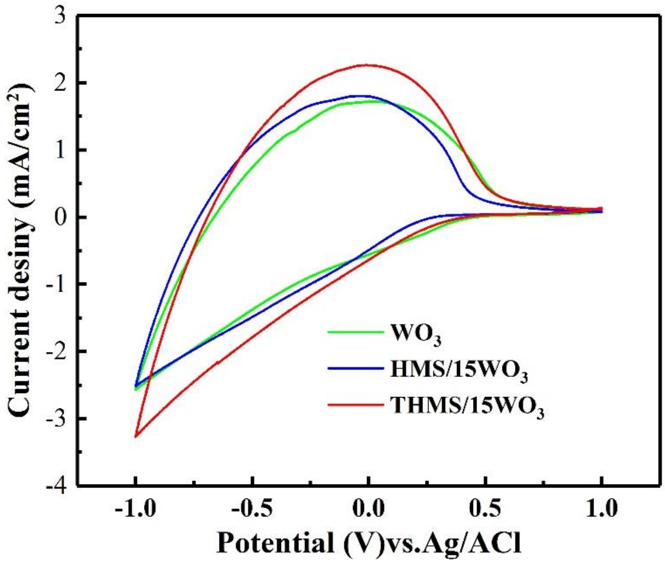

Fig. S2. Cyclic voltammograms of WO_3, THMS/15WO_3 and HMS/15WO_3 films at a scan rate of 50 mV s^{-1}.

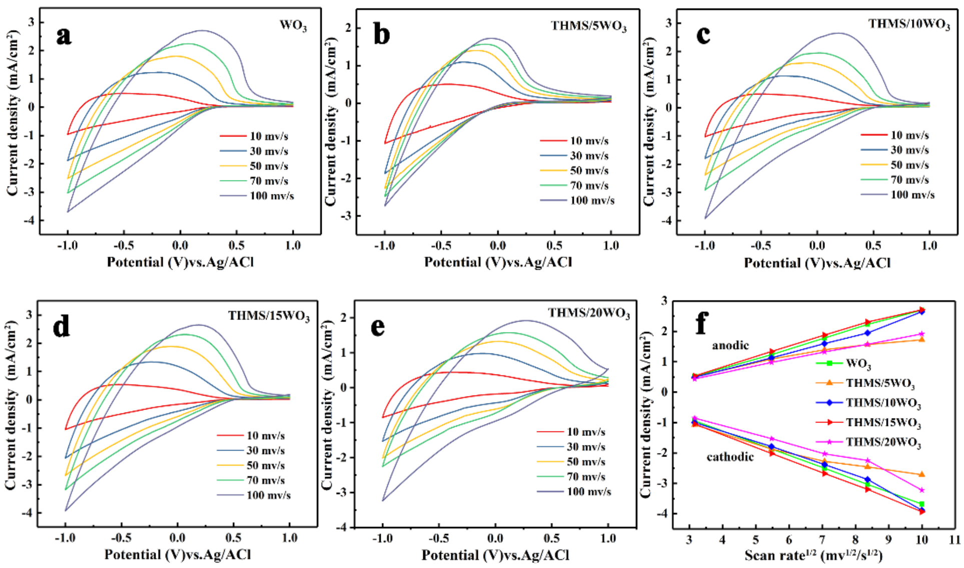

Fig. S3. CV curves of (a) pure WO_3, (b)THMS/5WO_3, (c)THMS/10WO_3, (d) THMS/15WO_3 and (e) THMS/20WO_3 hybrid films at different scan rates, (f) Plots of peak current density versus square root of scan rate for pure WO_3 and THMS/WO_3 films.
